# Supplementary material for: Intracellular delivery of mRNA to human primary T cells with microfluidic vortex shedding
Source: Sci Rep. 2019 Mar 1;9:3214. doi: 10.1038/s41598-019-40147-y (PMC6397276; doi:10.1038/s41598-019-40147-y)
Supplement: Supplementary file 1 — Supplement File [file 41598_2019_40147_MOESM1_ESM.docx]

**Supplementary Appendix**

**Intracellular delivery of mRNA to human primary T cells with microfluidic vortex shedding**

Justin A. Jarrell**^1**^**, Amy A. Twite**^1**^**, Katherine H. W. J. Lau**^1^**, Moein N. Kashani**^1^**^,^**^2^**^,^**^3^**, Adrian A. Lievano**^1^**, Julyana Acevedo**^1^**, Craig Priest**^2^**^,^**^3^**, Jorge Nieva**^1,4^**, David Gottlieb**^1,5^**, and Ryan S. Pawell**^1^**

**Supplemental Methods, Figures, and Tables**

**Supplemental Methods**

Device design and fabrication

Experimental rig development

Pan T cell culture

EGFP mRNA delivery to primary T cells at difference concentration

Quantifying EGFP expression levels in T cell subsets

T cell activation profiling

Medium composition

**Supplemental References**

**Supplemental Figure 1.** Consistent EGFP expression through duration of *μVS* transfection

**Supplemental Figure 2.** Computational fluid dynamic simulation of an empty chip

**Supplemental Table 1.** P-values for cell viability and EGFP expression efficiency measurements from Figure 4

**Supplemental Methods**

**Device Design & Fabrication**

The microfluidic chips were produced with deep reactive ion etching (DRIE) and thermal bonding. Vertical sidewalls were a product of DRIE on fused silica and a bulk material bond was produced with a thermal bond (Figure 1b). This production method allowed for high operating pressures and the use of an optically-transparent fused silica substrate allowed for device inspection.

**SI Table 1** Summary of designed and fabricated device features

| Feature | Design | Fabrication | Difference |
| --- | --- | --- | --- |
| Flow cell height (*h_fc_*) | 40.0 µm | 40.9 µm | 2.3% |
| Post diameter (*d*) | 40.0 µm | 42.2 µm | 4.0% |
| Row pitch (*p_r_*) | 60.0 µm | 59.5 µm | -0.8% |
| Channel wall thickness (*t*) | 6.0 µm | 6.2 µm | 3.3% |

The use of semiconductor processes resulted in minimal variation between designed and fabricated device geometries. The device yield, or percentage of successfully fabricated devices, was typically 95%. This high yield is attributed to relatively simple device geometries. For example, the maximum fabricated feature aspect ratio was approximately 6.6. DRIE is frequently used to generate features with substantially higher aspect ratios^1^.

The devices were designed with inlet and outlet channels that created uniform flow conditions^2^ and improved cell recovery. In preliminary experiments, device designs with open or channel-free inlets and outlets were capable of intracellular delivery; however, cell recovery was significantly lower and cell debris or buildup on the upstream side of the post was observed. These preliminary device designs were also prone to clogging when used to process larger volumes of cells and at high cell densities. This clogging was attributed to mechanical lysis of cells after impacting a post.

The introduction of inlet and outlet channels substantially reduced the opportunity for this form of cell lysis along with related device clogging. For the specific design used herein, fabricated posts occupied approximately 68.2% of the flow cell width, whereas the fabricated inlet and outlet channel structures occupied approximately 10.3% of the same flow cell width. Cellular debris was still observed at the inlet of the channels after processing, but cell recovery rates remained high and the devices were less prone to clogging particularly at the cell densities evaluated in this study.

**Experimental Rig Development**

Microfluidic equipment is typically limited to low flow rates, small sample volumes, and low operating pressures. Thus, a custom test rig was developed to withstand the high pressures required to generate *μVS* within the microfluidic chip. Direct measurement of microfluidic flow rates was not feasible with readily available commercial equipment due to the (1) significantly high flow rates, (2) sensitive nature of primary T cells, and (3) short flow times. For example, a 400 μL sample containing 16 x 10^6^ cells mL^-1^, processed at an operating pressure of 120 PSI (8.2 atmospheres) resulted in a measured flow rate between 5 to 8 mL min^-1^ and a total sample flow time of approximately 3 seconds. Additionally, the brief sample flow time coupled with limited tubing lengths, between the mass flow meter and the chip fitting, meant artifacts caused by diffusion and permeation of compressed nitrogen through the tubing wall was negligible, relative to flow of nitrogen driving the sample through the tubing and chip. Direct microscopy imaging of *µVS* with the current experimental rig was not possible because of the flow rate, short cell residence time, and rapid transfection.

This purpose-built system allowed us to quantify flow rates without disrupting (1) the hydrodynamic conditions within the chip or (2) the sample flow through the tubing. During preliminary experiments, it was observed that significant bends and kinks in the tubing would adversely affect cell viability and recovery – this was attributed to high shear recirculation regions created within the deformed tubing. Careful selection of tube inner diameter, length and material were required to generate the ideal flow conditions. Small changes in the inner diameter of the tubing resulted in significant changes in volumetric flow rates and hydrodynamics conditions within the chip, while excessive tubing lengths increased total sample loss. Cumulatively, an experimental rig was engineered to provide quantitative flow measurements of flow paths and subject cells to precisely-controlled hydrodynamic conditions within the microfluidic chip.

**Hydrodynamic Characterization**

Non-dimensional equations were used to calculate the Reynolds number (*Re*) in channels and flow around a cylindrical post^2^. This was done to determine if the post arrays were operating in the hydrodynamic conditions that support vortex shedding (*Re_o_ > 40*). The equations are based on the device’s volumetric flow rate (*Q*), kinematic viscosity of the fluid (ν) and specific device geometries.

Using non-dimensional analysis, the following Reynolds numbers were calculated: flow cell (*Re_fc_*), inlet channels (*Re_c_*), gap between posts (*Re_g_*), and flow around an object where the object is a cylindrical post (*Re_o_*). This allowed us to quickly and reasonably assess (1) if flow conditions were hydrodynamic and (2) the most probable cause of hydrodynamic conditions. *Re_fc_, Re_c_,* and *Re_g_* were calculated as follows:

$${Re}_{fc}=\frac{2Q}{\nu\left( h_{fc}+w_{fc} \right)}$$

$${Re}_{c}=\frac{2Q}{n\nu\left( h_{fc}+w_{c} \right)}$$

$${Re}_{g}=\frac{2Q}{n\nu\left( h_{fc}+w_{g} \right)}$$

Where *Q* is the device volumetric flow rate, *h_fc_* is the height of the flow cell, and *ν* is the kinematic viscosity of the fluid; the respective widths of the flow cell, channel, and gap are *w_fc_*, *w_c_*, and *w_g_*; and *n* is the number of gaps or channels and is calculated by:

$$n=\frac{w_{fc}}{p_{r}}$$

Where *p_r_* is the row pitch. *Re_o_* was calculated as follows:

$${Re}_{o}=\frac{\nu_{\infty}}{\nu}$$

where *v∞* is the free stream velocity and *d* is the post diameter. *v*∞ was calculated as follows:

$$\nu_{\infty}=\frac{Q}{h_{fc}w_{fc}}$$

*Re_o_* was then used to calculate the Strouhal number (*St*) for a smooth cylinder^2^ using the following equations:

$$St=0.21\left( 1-\frac{21}{{Re}_{o}} \right) 40< {Re}_{o}<200$$

$$St=0.198\left( 1-\frac{19.7}{{Re}_{o}} \right) 250< {Re}_{o}<2 x {10}^{5}$$

*St* was then used to approximate the frequency (*f* ) of vortex shedding using the following equation:

$$f=\frac{St\nu_{\infty}}{d}$$

**Pan T cell Culture**

Primary human CD3+ T cells were negatively selected from cryopreserved primary blood mononuclear cells (PBMCs) of a single donor using standard bead-based techniques. For revival and culture, 5 x 10^6^ pan T cells cell were thawed and seeded in X-VIVO10 with gentamycin and phenol red (Lonza). The cells were activated using CD3/28 T cell activator solution (StemCell Technologies) and 100 IU mL^-1^ recombinant human IL-2 (PeproTech) on the day of thaw and cultured at 37 ˚C in 5% CO_2_. Pan T cells were expanded for 16 days with addition of medium and IL-2 (100 IU mL^-1^) every two to three days to maintain cell concentration at or below 1 x 10^6^ cells mL^-1^. All data was collected between days 17 and 24 post thaw and activation.

**EGFP mRNA delivery to primary T cells at different concentrations**

All solutions that were used on chip were filtered using 0.22 μm filtration prior to use to remove particulates that could lead to clogging. For on chip processing, T cells were removed from culture and pelleted via centrifugation (5 min at 300 xg). Supernatant was removed via aspiration and the cell pellet was resuspended in 1x Dulbecco’s phosphate buffered saline (DPBS, Gibco). Cell were subsequently pelleted via centrifugation and aspirated. Rinsed cells were resuspended in filtered processing medium composed of Immunocult-XF (StemCell Technologies), 25 mM trehalose dihydrate NF (JT Baker, VWR) and 5% v/v DMSO (Corning Cellgro, Fisher Scientific) at a cell concentration around 2.0 x 10^7^ cells mL^-1^. Cell concentration was enumerated using a Countess II automated cell counter (Invitrogen) and trypan blue dye exclusion to assess viability. Three replicate samples were evaluated for each condition. To make the samples, 6.4 x 10^6^ cells were removed from the stock cell suspension, placed in 1.5 mL tubes, and diluted with processing medium to a final volume of 400 μL minus the mRNA volume. mRNA was added immediately before processing for 1.6 x 10^7^ cells mL^-1^ final cell concentration. Samples were filtered in two separate batches (160 μg mL^-1^ mRNA control, 10 μg mL^-1^, 40 μg mL^-1^, and 80 μg mL^-1^ as the first set of samples, handling control, 120 μg mL^-1^, 160 μg mL^-1^ and no mRNA device processed control as the second set) to reduce cell clumping, cluster formation, and potential for chip clogging.

The sample rig and tubing were sterilized before use with 70% ethanol wipe down and flush between each run. Prior to processing, each sample was mixed with the appropriate volume of EGFP mRNA (996 nt translated into a 26.9 kDa protein, 1 mg mL^-1^ TriLink BioTechnologies, San Diego CA, L-7601) at final concentrations ranging from 10 μg mL^-1^ to 160 μg mL^-1^ (30 nM to 473 nM). Samples were mixed thoroughly by pipetting and the sample tube was mounted in the tube fitting and the fitting clamped. Cell and mRNA suspension was then exposed to 120 PSI nitrogen pressure to drive the sample through the chip and induce intracellular mRNA uptake via *μVS* (Figure 1a). Processed samples were collected in 15 mL conical tubes and placed on ice until for the duration of the experiment to sync expression time points between samples. After each run, the rig and tubing were flushed with 70% ethanol and a new microfluidic chip was placed in the rig. Time equals zero for mRNA expression started when all samples were processed, removed from ice, and returned to culture medium. All samples within a set were processed within 30 min and the total time on ice for the samples ranged from 0.5 to 3.75 hrs. Control samples were performed in triplicate and remained at room temperature while the experimental samples were processed. Control samples that were not processed through the device consisted of 1.6 x 10^7^ cells mL^-1^ in processing medium (handling control), which was used to normalize the cell viability and recovery for the experimental samples, and in processing medium containing 160 μg mL^-1^ mRNA (mRNA control). Additional device control samples were set up at 1.6 x 10^7^ cells mL^-1^ in processing medium and ran through the device to determine the impact of *μVS* on cell survival without additional external factors (processing control).

After the last sample was processed and incubated on ice for 5 min, all samples were removed from ice, re-suspended, and an aliquot was taken from each sample for post processing cell viability and concentration quantitation. The remaining cells were diluted 1:20 in X-VIVO10 at a concentration of 8 x 10^5^ cells mL^-1^ with 100 IU mL^-1^ IL-2. Cultures were added to 6 well non-TC treated plates and cultured at 37 ˚C in 5% CO_2_ for growth, activation marker, and EGFP expression analysis at later times. Cell viability was monitored using trypan blue dye exclusion and growth was quantified using the Countess II cell counter. Additional IL-2 was added on days 2 and 4 after transfection and the cells were discarded on day 7, at a culture age of 24 d post thaw and activation.

Initial T cell viabilities and post processing concentrations were quantified by counting a minimum of two aliquots from each sample. Counts were used to determine the recovery and yield shown in Figure 2a. Cell growth and viability of each sample in growth medium was monitored over seven days (Figure 3a-b). EGFP expression and persistence at different time points post transfection were monitored using flow cytometry (Attune NxT flow cytometer) and propidium iodide (1 μM final concentration, Sigma Aldrich) to exclude dead cells (Figure 3c).

**Quantifying EGFP expression levels in T cell subsets**

Expression efficiency was examined among CD4+ and CD8+ T cell subsets using fluorescently-labeled monoclonal antibodies and flow cytometry. Cultures were analyzed 27 hr after being returned to culture medium. Aliquots were collected from 10, 80, and 160 μg mL^-1^ cultures, placed in a conical bottom 96 well plate, diluted with DPBS, pelleted via centrifugation, and aspirated. Cells were resuspended in 100 μL mL^-1^ of 25 μL mL^-1^ each mouse anti-human CD3-Alexafluor700 (ThermoFisher PN 56-0037-42), mouse anti-human CD4-PE-Cy7 (ThermoFisher PN 25-0048-42), and mouse anti-human CD8a-Super Bright 600 (ThermoFisher PN 63-0088-42) in DPBS containing 1% bovine serum albumin and 2 mM EDTA (flow buffer). Antibody staining was used to quantify the percentage of CD4+ and CD8+ T cells expressing EGFP. Cells were incubated in the antibody cocktail on ice for 30 minutes, then diluted with 100 μL flow buffer, pelleted via centrifugation, and aspirated. Samples were resuspended in 200 μL flow buffer, pelleted via centrifugation, and aspirated a second time before resuspending in 200 μL flow buffer and analyzed via flow cytometry. The experiment was compensated using a combination of AbC compensation beads (ThermoFisher) labeled with each antibody and EGFP-expressing cells (BL1 channel).

**Medium composition**

The impact of exposing human PBMCs to DMSO for long periods of time in culture conditions (37 ˚C, 5% CO_2_), was previously described by de Abreu Costa *et al.^3^* Briefly, long term exposure to various concentrations of DMSO resulted in increased cell death, decreased proliferation, and reduced cytokine production with increasing concentrations of DMSO and long exposure times (>4 h), after activation with phorbol-12-myristate-13-acetate (PMA)^3^. In our study, we showed that DMSO at 5% v/v did not impact cell viability until PBMCs were incubated in this solution for greater than 24 hrs at 37 ˚C and 5% CO_2_. Cytokine production in T cells after activation with PMA was negatively impacted by exposure to 5% DMSO but required eight hours of incubation time to show an effect. The max time for samples in processing medium was under four hours, and the samples were held on ice. The cells were processed in the order of lowest to highest mRNA concentration meaning 10 µg mL^-1^ spent the longest time on ice exposed to DMSO post processing. This could be why the growth rate of this group of cells was reduced post processing compared to the other groups, meaning that this reduction in growth rate is an artifact due to the experiment length. The decrease in viability of all groups compared to the handling control may be due to increased cell death upon return to culture due to unsuccessful membrane repair attempts, bulk mechanical lysis along with thermal shock, and medium stress, but this viability decrease is small when considering the device processed control.

Processing medium ionic strength may also have an impact on the overall ease of pore formation and cell recovery after *μVS* exposure. We observed a trend of decreasing viability with increasing mRNA concentrations, though this decrease was slight. This was potentially due to the higher percentage of mRNA added to samples containing higher concentrations of mRNA. For example, a 10 μg mL^-1^ sample contained 1% v/v mRNA buffer in the processing medium, while 160 μg mL^-1^ sample contained 16% v/v mRNA buffer. The mRNA solution is at a low ionic strength buffer (1 mM sodium citrate) relative to Immunocult-XF. This indicated that the reduction in total processing medium ionic strength associated with increasing mRNA concentration is a likely cause of decreased viability. The presence of mRNA in solution also decreased the concentration of trehalose, which could also result in lower cell viability with increasing mRNA concentration. Exploration of these variables, as well as the addition of concentrated buffers to adjust ionic strength, are currently ongoing.

**Supplemental References**

1. Marty, F., *et al.* Advanced etching of silicon based on deep reactive ion etching for silicon high aspect ratio microstructures and three-dimensional micro-and nanostructures. *Microelectronics Journal*, ***36*.7**, 673-677 (2005)
2. Gerhart, P. M., Gerhart, A. L., & Hochstein, J. I. Munson. Young and Okiishi's Fundamentals of Fluid Mechanics. *John Wiley & Sons*. (2016).
3. de Abreu Costa, L., *et al.* Dimethyl Sulfoxide (DMSO) Decreases Cell Proliferation and TNF-α, IFN-γ, and IL-2 Cytokines Production in Cultures of Peripheral Blood Lymphocytes. *Molecules*, ***22*.11**, 1789 (2017).

**Supplemental Figures**

**Supplemental Figure 1**. Consistent EGFP expression through duration of *μVS* transfection. Levels of EGFP protein expression were quantified in human T cells 25 hours post transfection with 80 μg mL^-1^ EGFP mRNA via *μVS*. Fractions represent post-processing cell samples collected at various time points from the start (Fraction 1) and end (Fraction 9) of the experiment. Data represent the mean ± SD of triplicate values. P values were calculated between groups by one-way ANOVA and post-hoc Kruskal-Wallis multiple comparisons test.

**Supplemental Figure 2.** Computational fluid dynamic simulation of an empty chip. Simulation of the flow dynamics in a 960μm width x 6.95mm length chip without posts depicting (a) velocity magnitude (m s^2^), (b) velocity vector (m s^2^), and (c) Q-criterion. Flow direction was simulated from left to right.

**Supplemental Table**

**Supplemental Table 1.** P-values for cell viability and EGFP expression efficiency measurements from Figure 4.
